# Patent ductus arteriosus, bronchopulmonary dysplasia and pulmonary hypertension—a complex conundrum with many phenotypes?

**DOI:** 10.1038/s41390-023-02578-2

**Published:** 2023-03-18

**Authors:** Afif EL-Khuffash, Rachel Mullaly, Patrick J. McNamara

**Affiliations:** 1 The Rotunda Hospital, Dublin, Ireland; 2 Department of Paediatrics, Royal College of Surgeons of Ireland, Dublin, Ireland; 3 Department of Pediatrics, University of Iowa, Iowa City, IA USA

The association between patent ductus arteriosus (PDA), bronchopulmonary dysplasia (BPD) and chronic pulmonary hypertension (cPH) in extremely low gestational age neonates (ELGANs) is an area gaining considerable interest. This triad of entities are all intertwined in a complex and multifactorial relationship. PDA can contribute to the development of BPD by increasing pulmonary blood flow, leading to lung injury and inflammation. Additionally, both PDA and BPD can contribute to the development of cPH by increasing pulmonary blood flow and vascular resistance leading to right ventricular dysfunction. The paper by Nawaytou et al., bravely attempts to make sense of it all. The authors attempted to divide PH associated with BPD into a predominantly flow based PH (associated with PDA left to right shunting) and PVR based PH (associated with vascular maladaptive changes). They reported a distinct association between the duration of PDA exposure and the former but not the latter.

The authors conclude that a “moderate/large PDA” increases the risk of flow associated cPH in the setting of BPD but found no association between the presence of a PDA and cPH resulting from pulmonary vascular disease (PVD). They postulated that prolonged exposure to a PDA does not result in pulmonary vascular remodeling prior to discharge from hospital. The authors argue that PVD “may be a manifestation of events occurring in utero or shortly after birth rather than a consequence of prolonged exposure to PDA”. The approach to this complex problem described in this paper is commendable, but there are other factors that warrant consideration. The findings of this study contradict a commonalty held belief of the impact a PDA has on pulmonary vasculature. Animal pulmonary overcirculation models clearly demonstrate an increased pulmonary artery pressure secondary structural and functional changes to the pulmonary artery media manifest as thickening of the walls and altered vascular responses to vasodilators. This is also supported in pathological models of post-mortem examinations of premature infants with large PDAs.^1–3^

There are several points worth further exploring in this paper. First, defining both the exposure (PDA) and outcomes (Flow versus PVD related PH) are fraught with difficulty using echocardiography. In terms of defining the exposure of interest, one of the major challenges facing neonatologists with haemodynamic expertise is characterising haemodynamic significance associated with a PDA (and accurately ascribing it as a disease state rather than an innocent bystander). Of note, PDA diameter in isolation is a poor predictor of PDA-related morbidities and as a result, attempting to study the relationship between a PDA and pulmonary arterial hypertension requires an accurate and validated assessment of haemodynamic significance. Almost every trial of PDA treatment, based on limited adjudication of hemodynamic significance, has showed no benefits to treatment.^4^ Therefore, interrogation of the complex and dynamic nature of the relationship between a hemodynamically significant PDA shunt and pulmonary vascular disease requires a prospective study design. In addition, the impact of changing loading conditions to the right ventricle over time and pressure loading of the left heart are important additional considerations. *Second*, the authors state in the introduction that *Pressure* = *Flow x Resistance*. This formula is incomplete as it ignores the contribution of pulmonary capillary wedge pressure (PCWP) to the whole relationship, which is very relevant in the setting of both PDA and BPD. PVR is calculated using the following formula: PVR = (PAP-PCWP)/Flow. The latter is an important consideration in the setting of prolonged exposure to a PDA as *Pressure* is the product of *Resistance* and *Flow* + pulmonary capillary wedge pressure; specifically, it is plausible that the elevation in pressure may occur due to an increase in one or all of these components. ELGANs universally exhibit left ventricular diastolic dysfunction leading to an increased left atrial pressure and a relatively high PCWP. These changes may be further exaggerated in the setting of PDA, where both ongoing respiratory distress and diastolic dysfunction were seen more commonly in babies with a PDA.^5^ Therefore, any echocardiography based definition of PH in this population should consider both pulmonary arterial and venous components and incorporate an assessment of LV diastolic dysfunction and left atrial pressure as they both have important clinical implications.^6^ The authors speculate that the abolition of elevated pressure following PDA closure relates to a reduction in the flow-driven component; while this is very plausible, it is also possible that there may be an improvement in post-capillary hemodynamics with reduced pulmonary venous and subsequently arterial pressure.

Regarding the outcome, the diagnosis of PH was made “if the tricuspid regurgitant jet velocity was >2.9 m/sec, the PDA systolic flow velocity estimated a peak systolic pulmonary artery pressure >35 mm Hg, or if systolic septal flattening was present (based on end-systolic eccentricity index >1.0).” The last criterion mentioned is arguably the most convincing for the present of PH. A TR jet is notoriously poor at estimating right ventricular systolic pressure in preterm infants. Its absence does not rule out the presence of PH and the jet can grossly under-estimate the true value in the presence of RV systolic dysfunction. The estimation of pulmonary systolic pressure using PDA flow can also be marred with difficulty as PDA diameter can play a significant role in determining velocity (in addition to pressure gradient). As such, it is very conceivable that many infants with true PH were missed from this cohort. Their criteria for diagnosing PVD related PH included the presence of PH in the absence of a PDA or the presence of PH in the alongside a non-significant PDA. Flow related PH was only diagnosed if a PDA was present and if PH disappeared after closure of the PDA. One of the additional limitations of the use of septal flattening (or end-systolic eccentricity index) is that it is entirely dependent on the interventricular pressure gradient. Therefore, in patients with systemic hypertension, which is being recognized with increased frequency in the setting of BPD, the septum may remain round and end-systolic eccentricity index <1.3 potentially leading to underestimation of pulmonary arterial pressure. Time dependent indices, such as pulmonary artery acceleration time have been shown to predict later development of PVD.^7^ While we agree that PDA-attributable flow driven PH was a major component in the BPD-PH seen, it would be premature to conclude that immediate resolution of PA pressure upon PDA closure is evidence of immunity to later or progressive PVD. It is concerning that 30% of the patients with BPD-PVD died, which highlights the seriousness of this condition. Prior reports of infants discharged with a PDA have reported PH-related mortality after discharge which emphasizes the progressive nature of this disease and the need for long-term monitoring.^8^ Longitudinal and standardized echocardiography evaluation of patients after PDA closure over time is therefore required to determine the true impact to both pulmonary vascular development and right ventricular performance. Without these data, it is not yet possible to conclude with certainty the life-long impact of prolonged PDA exposure to the immature heart and pulmonary vascular bed.

While the authors provide important and novel data on the relationship of prolonged PDA exposure to pulmonary vascular development, their data is a perfect illustration of the complexity of the premature infant’s cardiovascular and respiratory systems. Clinicians should be alerted to the fact that under the umbrella of “BPD” exists many distinct phenotypes (lung parenchymal phenotype, pulmonary vascular phenotype, flow driven phenotype, post-capillary phenotype and an admixture of all) which require an individualistic approach to monitoring and treatment. It is unlikely that a truly significant PDA with chronic left to right shunting plays no role in vascular remodeling over the first few weeks of age. Going forward, the neonatal haemodynamic community should continue to enhance standardization of definitions of a haemodynamically significant PDA, particularly in the setting of clinical trials, and prospectively evaluate the impact of prolonged exposure on the immature pulmonary vascular bed, right ventricle, and left heart.
